# Characteristics and trends in required home care by GPs in Austria: diseases and functional status of patients

**DOI:** 10.1186/1471-2296-7-55

**Published:** 2006-10-01

**Authors:** Gustav Kamenski, Waltraud Fink, Manfred Maier, Ingrid Pichler, Sonja Zehetmayer

**Affiliations:** 1 Department of General Medicine (Head: Manfred Maier), Centre of Public Health, Medical University Vienna, Währingerstrasse 13a, A-1090 Wien, Austria; 2 Section of Medical Statistics, Medical University Vienna, Spitalgasse 23, A-1090 Wien, Austria

## Abstract

**Background:**

Almost all societies carry responsibility towards patients who require continuous medical care at home. In many health systems the general practitioner cooperates with community based services of home care and coordinates all medical and non medical activities. In Austria the general practitioner together and in cooperation with relatives of the patient and professional organisations usually takes on this task by visiting his patients.

This study was carried out to identify diseases that need home care and to describe the functional profile of home care patients in eastern Austria.

**Methods:**

Cross sectional observational study with 17 GP practices participating during 2 study periods in 1997 and in 2004 in eastern Austria. Each GP identified patients requiring home care and assessed their underlying diseases and functional status by filling in a questionnaire personally after an encounter. Patients in nursing homes were excluded. Statistical tests used were t-tests, contingency tables, nonparametric Wilcoxon signed rank sum test and Fisher-combination test.

**Results:**

Patients with degenerative diseases of the central nervous system (65%) caused by Alzheimer's disease and cerebrovascular occlusive disease and patients with degenerative diseases of the skeletal system (53%) were the largest groups among the 198 (1997) and 261 (2004) home care cases of the 11 (1997) and 13 (2004) practices. Malignant diseases in a terminal state constituted only 5% of the cases. More than two thirds of all cases were female with an average age of 80 years. Slightly more than 70% of the patients were at least partially mobile.

**Conclusion:**

Home care and home visits for patients with degenerative diseases of the central nervous and skeletal system are important elements of GP's work. Further research should therefore focus on effective methods of training and rehabilitation to better the mental and physical status of patients living in their private homes.

## Background

Most societies carry responsibility towards patients requiring continuous medical attention and care at home [1-4]. In Austria the general practitioner together and in cooperation with relatives of the patient and professional organisations usually takes on this task by visiting his patients [5,6]. These patients need support for their chronic diseases such as sticks, absorbent pads or catheters, measures to prevent decubital ulcers or physiotherapy to improve their functional status, irrespective of whether they live in nursing homes or in their private homes [7,8]. An appropriate care for this group of patients increases the workload for the practice staff with regard to administration and organisation of all these home care related activities [9].

Detailed information about the diseases and the functional status of homecare patients not living in nursing homes is not available in Austria and there are no studies on the improvement of functional ability of the elderly population in the primary care setting. It was therefore the primary goal of this study to assess patients requiring homecare with regard to the disease(s) considered to be responsible for the need of homecare in the opinion of their GP. As secondary goals we tried to evaluate patients' functional status, the number of the physician-patient contacts as well as the number of care contacts of relatives and health care professionals with the patient. Furthermore, for the numbers available, we assessed the patients' degree of severity of illness according to the official grading system in Austria. The main goal of this study was to identify the diseases causing the need for home care and to evaluate the functional status of patients who are cared by GPs, patients' relatives and professional health services in the private homes of the patients.

Delivering health care services to the population and controlling the health care system is considered to be a public task in Austria. More than two thirds of this system is funded through social insurance contributions and general tax revenue. By paying a monthly compulsory contribution (including also employers' contributions) to social health insurance funds, nearly 99% of the Austrian population are insured and acquire entitlements to treatment as set out in the current general security provisions. Austrians are free to choose their GP or specialist and there exists no gate keeping function of the GP [10].

## Methods

In a cross sectional design a total of 17 GP solo-practices of comparable size (each caring for approximately 2000 patients) participated in this observational study. Practices of this size are typical for most regions of Austria [10]. Participating practices are situated in and around Vienna within a radius of approximately 70 kilometres. So the study population consists of inhabitants of Vienna, but also of inhabitants of small towns and villages in rural regions. The study patients are living in small families or, if living alone, they are cared by family members who visit them in scheduled time intervals. The recruitment of GPs who regularly visit their patients, took place at various local district meetings by a personal invitation to take part in the study. There were 2 study periods, the first one in January and February 1997 and the second one in February, March and April 2004. 11 (1997) respectively 13 (2004) GP practices of the eastern part of Austria participated. 4 practices contributed only to the study period of 1997, while in 2004 6 new practices joined the group. 7 practices with identical GPs took part in both study periods.

In all participating practices, patients requiring homecare were identified and the underlying causal disease(s) as well as their functional status were assessed by the GP using a questionnaire [see Additional file 1]. Participating GPs were asked to fill in the questionnaire personally after an encounter with the patient, his relatives and a member of the professional home care organisation.

The questionnaire was designed to assess age and gender and other demographic data, the size of the GP practice, orientation of the patient and functional status, as well as the main reason for his need of homecare. In addition, the number of contacts between the patient and his GP as well as between those who cared for the patient and the practice were recorded based on the documentation system. In the questionnaire the official grading of patients requiring homecare according to Austrian legislation was also documented. This grading system is used within the Austrian health insurance system to provide financial support to the patient and is executed by a special physician employed by the health insurance company. It is based on the number of hours per month needed for the care of the patient. Grade 1 means a minimum of 50 hours and grade 7 means a maximum of 180 hours for the care and includes the total immobility of the patient.

All patients who were unable to regularly execute activities of daily life by themselves and therefore required the help of other people were included in the study. Patients living in nursing homes were excluded.

Data were assessed by the statistical programs EPI Info, Version 6 and SAS 8.

Methods used were t-tests for the comparison of age between women and men in 1997 and in 2004 and contingency tables with a chi-square test for the comparison of the degrees of the official grading system between 1997 and 2004. Here, p-values < 0.05 were considered as significant. For the comparison of the frequency of diseases between 1997 and 2004 on the one hand a nonparametric Wilcoxon signed rank test (paired analyses) for the practices taking part in both study periods and on the other hand a Wilcoxon rank sum test (unpaired analysis) for practices taking part in only one period was calculated for each disease group.

Then a Fisher-combination test was performed for the resulting independent p-values, that means the comparison between 1997 and 2004 was considered as significant if the product of the two p-values was < 0.00870 (two-sided test with an overall significance level 0.05) [11]

## Results

Between 18 and 20 patients per GP required homecare in both study periods.

On average the GP visited these patients 2.2 times per month in 1997 and 2.7 times per month in 2004. The number of contacts between the relatives or the nursing professionals and the GP practice was 1.8 times per month in 1997 and 1.4 times per month in 2004 (figures not shown in table).

Table 1 shows the number of participating practices, patients' characteristics and the degrees of the official grading system during the 2 study periods. 1997 as well as 2004 there was a significant difference in the age between men and women. In the degrees of the official grading system there was a tendency towards more patients in the group of the less severe cases (groups 1+2) in the year 2004. About 85% of the patients were taken care of by relatives in the year 1997 as well as 2004 while 14.2% (1997) and 38.1% (2004) of the patients were attended by nursing professionals.

**Table 1:** Number of practices, patients' characteristics and grading according to the official Austrian grading system

***Year***	***1997***	***2004***
Participating practices	N = 11	N = 13
Total number of patients	N = 198	N = 261
	n (%)	n (%)
Sex		
m	56 (28.3)	79 (30.3)
f	142 (71.7)	182 (69.7)
Mean age		
m/f	77.2 (SD 17.5)	79.9 (SD 13.7)
m*	72.1 (SD 18.2)	76.3 (SD 16.1)
f*	79.2 (SD 16.8)	81.4 (SD 12.2)
Married	56 (28.3)	76 (29.1)
Divorced	4 (2.0)	19 (7.3)
Widowed	110 (55.6)	139 (53.3)
Single	28 (14.1)	27 (10.3)
Former occupation		
Housewife	81 (40.9)	96 (36.8)
Self-employed	41 (20.7)	46 (17.6)
Employed	62 (31.3)	106 (40.6)
None	14 (7.1)	13 (5.0)
		
Care provided by	†N = 197	†N = 257
Relatives	169 (85.8%)	218 (84.8%)
Professional health care services	28 (14%)	83 (38.1%)
		
Degrees of the official grading system	Number of patients (attached to degree 1–7)	
	n (%)	n (%)
1+2‡	43 (24.7)	109 (51.9)
3+4	103 (59.2)	85 (40.5)
5+6+7	28 (16.1)	16 (7.6)
	†174 (100.0)	†210(100.0)

*1997 as well as 2004 there was a significant difference in the age between men and women [p = 0.0093 (1997); p = 0.014 (2004)] in the t-test.

†Assessment was not available in all cases of the years 1997 and 2004

‡Compared with the year 1997 there was a tendency towards more patients in the group of the less severe cases (degree 1+2) in the year 2004 (p < 0.0001) in the official grading system.

Table 2 shows the functional status of the patients requiring home care and compares the assessment made in 1997 with the data from the second study period in 2004.

**Table 2:** Functional status of patients of all practices in the years *1997 *(N = 198) und *2004 *(N = 261)

***Year***	***1997***	***2004***	***1997***	***2004***	***1997***	***2004***
	Yes		In part		No	
	n	(%)*	n	(%)*	n	(%)*
Oriented(time)	116 (58.6)	182 (69.7)	31 (15.7)	51 (19.5)	51 (25.8)	28 (10.7)
Oriented(location)	143 (72.2)	213 (81.6)	26 (13.1)	33 (12.6)	29 (14.6)	15 (5.7)
Mobile(complete)	42 (21.2)	67 (25.7)	19 (9.6)	44 (16.9)	137 (69.2)	150 (57.5)
Mobile with support	85 (42.9)	148 (56.7)	26 (13.1)	46 (17.6)	87 (44.0)	67 (25.7)
Bedridden	45 (22.7)	27 (10.3)	29 (14.6)	39 (14.9)	124 (62.6)	195 (74.7)
Incontinent	97 (49.0)	91 (34.9)	26 (13.1)	56 (21.5)	75 (37.9)	114 (43.7)
Able to communicate	175 (88.4)	232 (88.9)	10 (5.1)	18 (6.9)	13 (6.6)	11 (4.2)
Able to dress by themselves	69 (34.8)	125 (47.9)	32 (16.2)	80 (30.6)	97 (49.0)	56 (21.5)
Personal hygiene	46 (23.2)	79 (30.3)	45 (22.7)	91 (34.9)	107 (54.0)	91(34.9)
Ability to eat by themselves	136 (68.7)	208 (79.7)	30 (15.2)	29 (11.1)	32 (16.2)	24 (9.2)
Ability to take medication by themselves	57 (28.8)	118 (45.2)	22 (11.1)	38 (14.6)	119 (60.0)	105 (40.2)
Danger of falls	155 (78.3)	185 (71.0)	19 (9.6)	22 (8.4)	24 (12.1)	54 (20.7)
						
**Frequency of home care visits (provided by relatives or health care professionals)**						
***Year***		***1997***		***2004***		
Number of home care visits per day		n (%)		n (%)		
1x/day		11 (5.8)		49 (20.3)		
2x/day		22 (11.5)		59 (24.5)		
3x/day		43 (22.5)		38 (15.8)		
Continuous care		115 (60.2)		95 (39.4)		
Sum		191†(100)		241†(100)		

*All percentage rates apply to the totality of cases N = 198 (*1997*) and N = 261 (*2004*)

†It was only possible to assess the number of home care visits in 191 of 198 patients (1997) and in 241 of 261 patients (2004)

The assessment of the functional status in 1997 shows that 69% were not totally mobile, 43% were mobile only with some support, and 23% were bedridden. About 50% of the patients could not dress themselves or take care of their hygiene.

There was a trend in direction of an improvement of the most of the functional parameters in 2004.

Table 3 summarizes the underlying diseases mainly responsible for the need for homecare of the patients. As can be seen diseases of the central nervous and of the skeletal system were the main reasons for requiring home care. Diseases of the central nervous system were responsible for 67% (1997) and 63% (2004) of all home care cases. Diseases of the joints and the vertebral column significantly increased from 42% to 65% in 2004.

**Table 3:** Groups of diseases causing the need for home care

***Year***	***1997***	***2004***
	N = 198	N = 261
**Diseases of the CNS**	n = 133 (67.2%)	n = 165 (63.2%)
*ICD 10: F00, F01, G20, G30, G31, G35, G46, G80, G81, G83, I61, I63, I64, T90	(unpaired p = 0.83, paired p = 0.22, combination p = 0.18)	
		
**Diseases of joints and vertebra†**	n = 84 (42.4%)	n = 168 (64.9%)
*ICD 10: M05, M06, M15, M16, M17, M42, M47, M51, M53, M80	(unpaired p = 0.03, paired p = 0.08, combination p = 0.002)	
		
**Diseases of the cardiovascular system**	n = 78 (39.4%)	n = 102 (39.2%)
*ICD 10: I08, I11, I20, I25, I42, I50, J42, J43, J45	(unpaired p = 0.75, paired p = 0.94, combination p = 0.7)	
		
**Metabolic diseases‡**	n = 30 (15.2%)	n = 60 (23.0%)
*ICD 10: E10, E11, K71, K74, N18	(unpaired p = 0.59, paired p = 0.03, combination p = 0.019)	
		
**Other diseases**	n = 64 (32.2%)	n = 63 (24.2%)
*ICD10: C00–C97, E41, E64, H54, H80	(unpaired p = 0.34, paired p = 0.69, combination p = 0.23)	

*To point out the most frequent diseases of each group as they are coded by the International Classification of Diseases (ICD 10) system.

†Significant difference in the frequency of the diseases of joints and vertebra between 1997 and 2004.

‡There is a trend of an increasing frequency of metabolic diseases between 1997 and 2004.

Cardiovascular diseases constituted 39% of the cases requiring home care in both periods.

The percentage of patients requiring home care who suffered from the complications of long lasting diabetes increased from 11% (1997) to 19% (2004).

The group of the other diseases consisted of 32% (1997) and 24% (2004) of all cases.

Table 4 Among the diseases of the central nervous system, degenerative disorders (mainly dementia of Alzheimer's type) followed by vascular reasons were leading problems. Among diseases of the joints and the vertebral column, again degenerative problems were the main reason for requiring homecare. Coronary artery disease and cardiac insufficiency were the main reasons among the diseases of the cardiovascular system. Among metabolic diseases diabetes was the leading cause for the requiring of home care. Among the other diseases senile marasmus (chronic dehydration and malnutrition not caused by certain disease) was the leading cause. Malignancies in the end stage played at least quantitatively a minor role in both investigation periods.

**Table 4:** Diseases causing the need for home care in detail

***Year***	***1997***	***2004***
**Central nervous system**	**N = 133 (67.2%)**	**N = 165 (63.2%)**
		
Dementia degenerative	n = 65 (32.8%)	n = 97 (37.2%)
vascular	n = 57 (28.8%)	n = 80 (30.6%)
Congenital	n = 11 (5.6%)	n = 8 (3.1%)
Toxic (hepatic, uremic, ethylic.)	n = 7 (3.5%)	n = 14 (5.4%)
Inflammatory (status post.)	n = 5 (2,5%)	n = 5 (1.9%)
Epileptic	n = 4 (2%)	n = 8 (3.1%)
Posttraumatic	n = 4 (2%)	n = 7 (2.7%)
		
**Joints and vertebra**	**N = 84 (42.4%)**	**N = 168 (64.4%)**
		
Degenerative	n = 71 (35.8%)	n = 153 (58.6%)
Posttraumatic	n = 11 (5.6%)	n = 25 (9.6%)
Inflammatory (status post)	n = 6 (3.0%)	n = 24 (9.2%)
Congenital	n = 3 (1.5%)	n = 5 (1.9%)
		
**Cardiorespiratory and vascular system**	**N = 78 (39.4%)**	**N = 102 (39%)**
		
Coronary heart disease and chronic heart failure	n = 70 (35.4%)	n = 84 (32.2%)
COPD und asthma	n = 11 (5.6%)	n = 30 (11.5%)
Peripheral arterial occlusive disease	n = 8 (4.0%)	n = 29 (11.1%)
		
**Metabolic diseases**	**N = 30 (15.2%)**	**N = 60 (23.0%)**
		
Diabetes	n = 22 (11,1%)	n = 49 (18.8%)
Hepatic	n = 6 (3.0%)	n = 10 (3.8%)
Renal	n = 7 (3.5%)	n = 7 (2.7%)
		
**Other diseases**	**N = 64 (32.3%)**	**N = 63 (24.1%)**
		
Marasmus senilis	n = 38 (19.2%)	n = 25(9.6%)
Blindness	n = 14 (7.1%)	n = 25 (9.6%)
Deafness	n = 14 (7.1%)	n = 18 (6.9%)
Terminal malignant disease	n = 10 (5.1%)	n = 11 (4.2%)

Note: All percentage rates apply to the totality of cases N = 198 (*1997*) and N = 261 (*2004*)

## Discussion

As far as we are aware this is the first survey of patients requiring home care from the perspective of the GP in Austria. As an essential result of the study it turned out that the focal point in the management of the diseases of GPs' home care patients is not the cancer patient in the terminal stage, but the patient with degenerative diseases of the CNS and the musculo-skeletal system.

About 85% of the patients were cared for by one or more of their relatives. 2/3 of the patients were female and 5 years older than males. This correlates with the higher life expectancy for women [12,13].

The number of 2.2 (1997) and 2.7 (2004) home visits per month, was rather low considering the number of coexisting diseases. A possible explanation for this result could be that regular home visits by the GP and other health care professionals support the relatives providing care, foster the health status of patients and help to prevent deterioration [14].

Concerning the change in the proportion that has permanent home care, going from 60% 1997 to 40% in 2004 we do not believe that this change is due to changed living arrangements, as these did not change appreciably in this area. Elderly people, as mentioned above, usually live alone (regularly visited by their children or other relatives living close by) or in small families. Also 1997 it was already very infrequent that elderly people lived in extended families and this situation did not change until now.

A possible explanation for the reduction of elderly people needing permanent care could be that the number of nursing homes has increased and that access to nursing homes is more easy and better accepted [10,13].

Comparing the functional parameters between 1997 and 2004 shows a trend in the direction of an improvement in most of the parameters assessed. The improvement in the functional parameters parallels the decrease in the severity of the degrees of the official Austrian grading system. This trend could indicate that patients with more serious medical problems are kept in nursing homes, as we found evidence for an increasing trend (a plus of 20,3% between 1997 and 2004) for institutionalization of elderly people in this area with a rate of 126 for every 1000 people aged 75 or older [10,13]. A more optimistic interpretation could be that the continuous care by relatives, professionals and physicians improves functional parameters over time.

Diseases of the CNS constituting the majority of the cases remained equally frequent and cause a heavy burden for those who care for these patients. Two thirds of all patients show an orientation in time and location, which can be explained by the fact that certain diseases of the CNS (e.g. strokes, sclerosis, sequelaes of brain trauma, Parkinson's disease) do not regularly cause an impaired orientation, but are nevertheless impairing the patient enough to make him unable to care for himself [15-18].

The increasing number of the diseases of the musculo-skeletal system is not surprising, considering the general trend in civilized nations towards a high prevalence of musculo-skeletal disorders [19]. Obviously there exists a contradiction between the bettering of the functional parameters and the increasing number of musculo-skeletal diseases, which possibly can be explained by an increasing number of less severe cases despite the higher mean age of patients in the second observation period of 2004.

The tendency towards more severe cases of diabetes parallels the increasing prevalence of diabetes at the national [20,21] and international level [22].

Malignant diseases constituted only a small proportion of the diseases leading to the need for home care. Despite their low number, patients suffering from terminal malignant disease require special competence for example with regard to pain management. The cooperation between the patient, relatives, nursing professionals and palliative care teams is therefore of high importance [23-26].

The high prevalence of marasmus senilis (19 respectively 10%) compares well with the results of other studies which show figures between 5 and 37% [27,28].

One of the strengths of our study is that it gives a first insight in the underlying diseases leading to the need of home care and in the functional status of patients living in their private homes who are cared for there by their GPs. Another perhaps unexpected result is the high involvement of the relatives in the caring process in addition to professional care givers.

One of the main limitations of our study is the small number of practices in the restricted geographical area of eastern Austria. Another limitation is that the practices of the two study periods were only partially identical. So the comparison of the results of the two periods could have been biased by this.

In accordance with the existing literature we found that degenerative diseases of the brain and the skeletal system are the main reason for chronic disability also in our group of home cared patients and that home visits play an important role in the process of continuous home care [1,5,6,8,12,17,19].

## Conclusion

Long term care and home care for patients with degenerative diseases of the central nervous and skeletal system are important elements of GP's work. Further research in general practice and family medicine should therefore focus on effective methods of training and rehabilitation to better the mental and physical status of this group of patients living in their private homes.

To contextualize the results of our geographically limited study and to examine if they are valid for the rest of Austria and the other countries of the EC, further research work is necessary, as the problem of an increasing number of elderly people requiring home and long term care affects many health care systems.

## Competing interests

The author(s) declare that they have no competing interests.

## Authors' contributions

GK, as the main author of the study initiated, discussed and organized the concept of the study from the beginning including acquisition, analysis and interpretation of data.

WF participated in the study by helping to draft the manuscript and to check the relevant literature.

MM contributed to the study by critically analyzing especially the data and conclusions of the first study period and by revising the concept, results and conclusions of the whole study.

IP participated especially in drawing up the first concept of the study and she helped also in building up the small research network of practices.

SZ was involved in the study by doing the statistics and by critically revising the final results and conclusions of the study.

All authors read and approved the final manuscript.

## Pre-publication history

The pre-publication history for this paper can be accessed here:



## Supplementary Material

Additional file 1 Study questionnaire. This questionnaire had to be filled in personally by the GP after an encounter. Click here for file
