# Supplementary material for: Characteristics and trends in required home care by GPs in Austria: diseases and functional status of patients
Source: BMC Fam Pract. 2006 Oct 1;7:55. doi: 10.1186/1471-2296-7-55 (PMC1592492; doi:10.1186/1471-2296-7-55)
Supplement: Additional file 1 — Study questionnaire. This questionnaire had to be filled in personally by the GP after an encounter. [file 1471-2296-7-55-S1.doc]

Characteristics and trends in required home care by GPs in Austria: diseases and functional status of patients

**Questionnaire**  Date yymmdd

(to be filled in by the doctor personally!)

Name of practice_________________ Practice size: -500:1 –1000:2 –1500:3 –2000:4 –2500:5 ____

# Patient`s second name_________________ First name_________________Age_________Sex___

Married (or long lasting relationship with a partner):1 Divorced:2 Widowed:3 Single:4_____

Education :Elementary school:1 School for education in a handcraft:2 Trade school:3 High school:4 University:5 No school education :6 ___ Community size: -10000:1 –20000:2 +20000:3___

Former occupation: Housewife:1 Self-employed:2 Employed:3 None:4____

Somatic/ psychic status Yes:1 No:2 In part:3

Oriented (time)__ Oriented (location)__ Able to communicate___

Manifest psychosis__ Complete mobile__ Mobile with support__ Bedridden__ Able to dress by themselves__

Personal hygiene__ Able to eat by themselves__ Able to take medication by themselves__Incontinent__ Danger of falls__

Personal safety__ Lives in his/ her own home__ Lives with relatives__ Home visited by a professional care organisation__ Supported by neighbourhood__ Visited regularly by relatives__

Doctor`s consultations/ month____ Assistant`s consultations/month ___ Frequency of daily visits of professional care providers and/or relatives (1-3; permanent:4)___

Degree of official grading system (0-7; none:0; unknown: empty entry)__ MMSE (0-30)__

Classification of the medical reason for the need of homecare Yes Y No N

Diseases of CNS

Degenerative (e.g. Alzheimer`s disease, Parkinson`s disease)__ Congenital __

Traumatic__

Toxic__ Vascular (e.g.stroke, cerebral occlusive disease)__

Inflammatory__ Epileptic__

Diseases of joints and vertebra

Traumatic__ Inflammatory__ Degenerative__ Congenital__

Metabolic diseases

Diabetes__ Hepatic__ Renal__

Cardiorespiratory and vascular system

Coronary HD, chronic heart failure__ COPD; Asthma__ Peripherial occlusive disease___

Other diseases

Terminal malignant disease__ Marasmen senilis__ Blindness__ Deafness__

Additional relevant diseases

Liver__ Pankreas__ Kidneys__ Lung__ Heart__ Diabetes

Impairment of seeing and hearing __ Hypertension__ CNS__ Hematologic diseases__

Diseases of vessels__ Gastroenterologic__ Neoplasma__ Prostatic__

Note: Diseases summarized under „Classification of medical reason for homecare“ are the main reason for the need of home care. Diseases summarized under “Additional relevant diseases” are not the cause of the need of home care, but seem to be important enough to be mentioned by the treating GP.
